# Nanodelivery of Natural Antioxidants: An Anti-aging Perspective

**DOI:** 10.3389/fbioe.2019.00447

**Published:** 2020-01-10

**Authors:** Alexander Vaiserman, Alexander Koliada, Alina Zayachkivska, Oleh Lushchak

**Affiliations:** ^1^Laboratory of Epigenetics, D.F. Chebotarev Institute of Gerontology, NAMS, Kyiv, Ukraine; ^2^Department of Biochemistry and Biotechnology, Vasyl Stefanyk Precarpathian National University, Ivano-Frankivsk, Ukraine

**Keywords:** age-associated disorders, nano-antioxidants, phytochemicals, oxidative stress, bioavailability, nanodelivery, anti-inflammatory properties

## Abstract

The aging process is known to be associated with heightened oxidative stress and related systemic inflammation. Therefore, antioxidant supplementation is regarded as a promising strategy to combat aging and associated pathological conditions. Food-grade antioxidants from plant-derived extracts are the most common ingredients of these supplements. Phyto-bioactive compounds such as curcumin, resveratrol, catechins, quercetin are among the most commonly applied natural compounds used as potential modulators of the free radical-induced cellular damages. The therapeutic potential of these compounds is, however, restricted by their low bioavailability related to poor solubility, stability, and absorbance in gastrointestinal tract. Recently, novel nanotechnology-based systems were developed for therapeutic delivery of natural antioxidants with improved bioavailability and, consequently, efficacy in clinical practice. Such systems have provided many benefits in preclinical research over the conventional preparations, including superior solubility and stability, extended half-life, improved epithelium permeability and bioavailability, enhanced tissue targeting, and minimized side effects. The present review summarizes recent developments in nanodelivery of natural antioxidants and its application to combat pathological conditions associated with oxidative stress.

## Introduction

Population aging is becoming an increasingly important social and economic challenge for modern society. This is because the rising human life expectancy is generally not accompanied by the same increase in healthspan. The burden of age-related disorders has been steadily increasing over the past decades in most developed countries (Seals et al., 2016; Yabluchanskiy et al., 2018). Therefore, the development of treatment modalities specifically targeting aging pathways becomes a vital task of medical research and healthcare industry now (Vaiserman and Marotta, 2016; Vaiserman and Lushchak, 2017; Myers and Lithgow, 2019). Development and implementation of such therapeutic options is a main goal of a new research platform, translational geroscience, aimed at optimizing and preserving physical and psychosocial functioning throughout the human lifespan and compress disabilities and morbidity into a shorter period of late-life to achieve optimal longevity (Seals and Melov, 2014; Seals et al., 2016). A progress in this rapidly developing field is certainly depended on unraveling the mechanistic basis of aging process (Crimmins, 2015). Over three last decades, there was an unprecedented growth in our understanding of the basic pathways underlying aging (Campisi et al., 2019). The aging-associated chronic oxidative stress and related systemic inflammation are undoubtedly among pathways most significantly contributing to cellular senescence and aging (Liguori et al., 2018). Damages in vital biomolecules, including lipids, proteins (enzymes) or nucleic acids, caused by reactive oxygen species (ROS) and other free radicals generated in mitochondria through metabolic processes are considered to be among the primary drivers of aging process (Lushchak et al., 2014; Santos et al., 2018). ROS have been repeatedly shown to be implicated in basic aging-associated processes such as telomere attrition (Barnes et al., 2019), autophagy (Pajares et al., 2018), and exhaustion of the stem-cell population (Chen et al., 2017). The roles of oxidative stress and related inflammation are also becoming increasingly evident in the pathogenesis of most age-related pathological conditions including atherosclerosis (Yang et al., 2017), insulin resistance (Yaribeygi et al., 2019), and various cardio-metabolic and neurodegenerative diseases (Verdile et al., 2015; Garaschuk et al., 2018; Cenini et al., 2019). Based on these theoretical considerations, reducing the oxidative stress level by dietary supplementation with antioxidants known to neutralize free radicals by electron donation is recognized by many authors as a promising interventional strategy to delay or prevent age-related pathological conditions.

The therapeutic potential of dietary antioxidants is, however, restricted in most cases by their low bioavailability related to their poor solubility and stability in gastrointestinal fluids. Currently, innovative nanotechnology-based applications aimed at improving the oral bioavailability, and, accordingly, therapeutic effectiveness of phytochemicals and other natural antioxidants are emerging. The aim of this review is to provide information about recent advances in the application of phytoantioxidant-based nanodelivery systems to combat the aging-related oxidative stress and associated pathological conditions.

### Synthetic Antioxidants: Health Benefits and Hazards

Chronic oxidative stress-associated ROS overproduction is known to lead to damage of vital biomolecules and abnormal expression of various genes, including those involved in aging pathways (Lushchak, 2014; Tan et al., 2018). Therefore, it is considered to be one of the most important contributing factors in onset and progression of many aging-related pathological conditions. Under normal physiological conditions, ROS levels are strongly controlled in the cell by endogenous antioxidant enzymes, such as superoxide dismutase (SOD), glutathione (GSH) peroxidase, and catalase (CAT), and by exogenous antioxidants, such as vitamins, minerals, and polyphenols (Rahal et al., 2014). Under age-related pathological conditions, abnormally large ROS concentrations can damage a wide variety of biomolecules, thereby resulting in permanently disrupted profiles of gene expression and signal transduction, thus accelerating aging (Tan et al., 2018). Chronic oxidative stress condition also leads to an enhanced level of non-enzymatic, covalent attachment of glucose molecules to proteins, resulting in an elevated generation of advanced glycation end products (AGEs), known to be substantially contributed to aging and age-associated disorders (Kim C. S. et al., 2017; Chaudhuri et al., 2018; Fournet et al., 2018). In addition, the glycation of antioxidant enzymes such as copper-zinc SOD and GSH reductase may impair the organism's endogenous antioxidant defense system (Fournet et al., 2018). Chronic oxidative stress may also result in the accelerated telomere attrition with age. Telomeres are nucleotide repeated sequences of eukaryotic linear chromosomes that cap the chromosome ends to protect from undesirable enzymatic activities. In absence of specific enzymatic activity of telomerase, telomeres shorten with every cell division, being thereby substantially involved in aging processes (Hayashi, 2018; Liu et al., 2019). Emerging evidence, however, indicates the age-related telomere shortening depends not only on the number of divisions that cells have undergone, but also on the level of oxidative stress (Koliada et al., 2015). Taken together, these considerations indicate that oxidative stress may significantly contribute to aging and associated diseases, suggesting that counteracting oxidative stress may be an effective way to slow down or delay aging (for reviews, see Liguori et al., 2018; Forni et al., 2019; Zuo et al., 2019).

**Figure 1:**
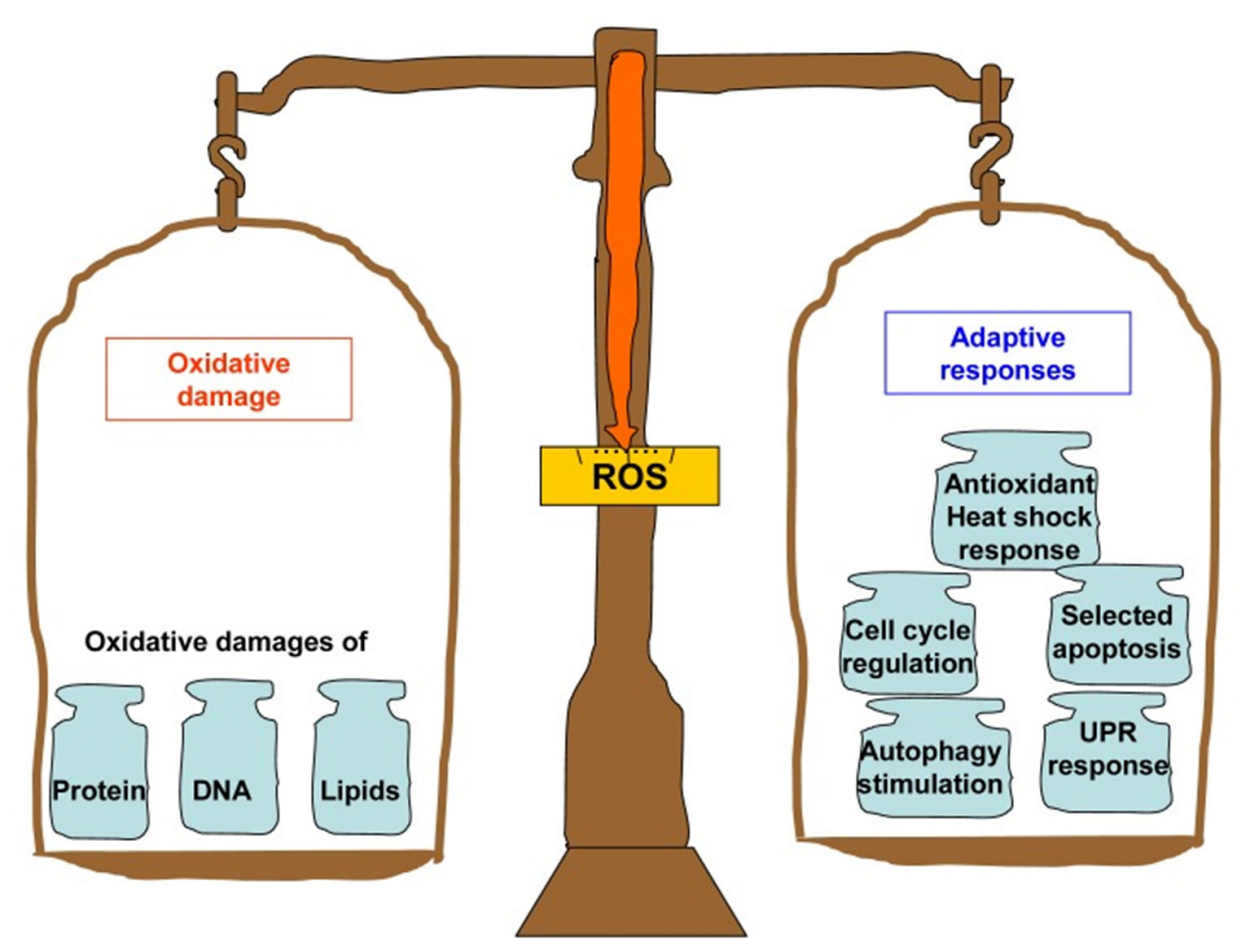
Summary of the bifurcated effects that can be induced by ROS. On the one hand, ROS induces the oxidative damage to proteins, DNA and lipids. On the other hand, they also trigger the organism's adaptive responses including antioxidant and heat shock responses, fatty acid deacylation-reacylation, cell cycle regulation, DNA repair and apoptosis, unfolded protein responses, and autophagy stimulation. The figure and its legend are reproduced from the open-access article by Mao and Franke (2013) distributed under the terms of the Creative Commons Attribution License with permission from the authors.

Thus, supplementation with dietary antioxidants is traditionally considered by many scholars and medical practitioners as a reasonable intervention strategy to delay or prevent age-related pathological conditions. The research findings on this topic are, however, quite controversial. On the one hand, supplementation with dietary antioxidants indeed caused beneficial effects on aging and longevity in various animal models (Sadowska-Bartosz and Bartosz, 2014). The popularization of these findings has led to the situation where more than half of adult residents of high-income countries regularly consume dietary antioxidants with hope to slow aging and extend healthspan (Bjelakovic et al., 2014). On the other hand, many randomized controlled trials of antioxidant supplements in humans have failed to improve health and prevent cardiovascular diseases and other age-related pathological conditions (Myung et al., 2013; Gruber and Halliwell, 2017). Furthermore, synthetic antioxidants such as vitamins were found to be potentially dangerous, and some epidemiological findings indicate that regular intake of β-carotene, vitamin A and vitamin E may even increase mortality, especially in well-nourished populations (Bjelakovic et al., 2014). The discrepancy between results obtained in animal models and clinical trials, which is generally referred to as an “antioxidant paradox” (Halliwell, 2000), may probably be due to the dual roles of antioxidants in ROS production. Indeed, dietary antioxidants can act not only as ROS scavengers; they may also be easily oxidized and act as pro-oxidants to induce damages in vital biomolecules when present in a large concentration (Milisav et al., 2018). Moreover, accumulating evidence suggests that ROS play crucial roles as important second messengers involved in many vital processes, including intracellular signaling, cell survival, proliferation, differentiation and apoptosis, and also immune and stress responses (Schieber and Chandel, 2014; Pizzino et al., 2017; Bardaweel et al., 2018; Milkovic et al., 2019; see also Figure 1 for illustration). The considerations above imply that targeting ROS-related disorders by pharmacologically-synthesized antioxidant drugs is extremely complex and difficult task.

### Natural Antioxidants

**Figure 2:**
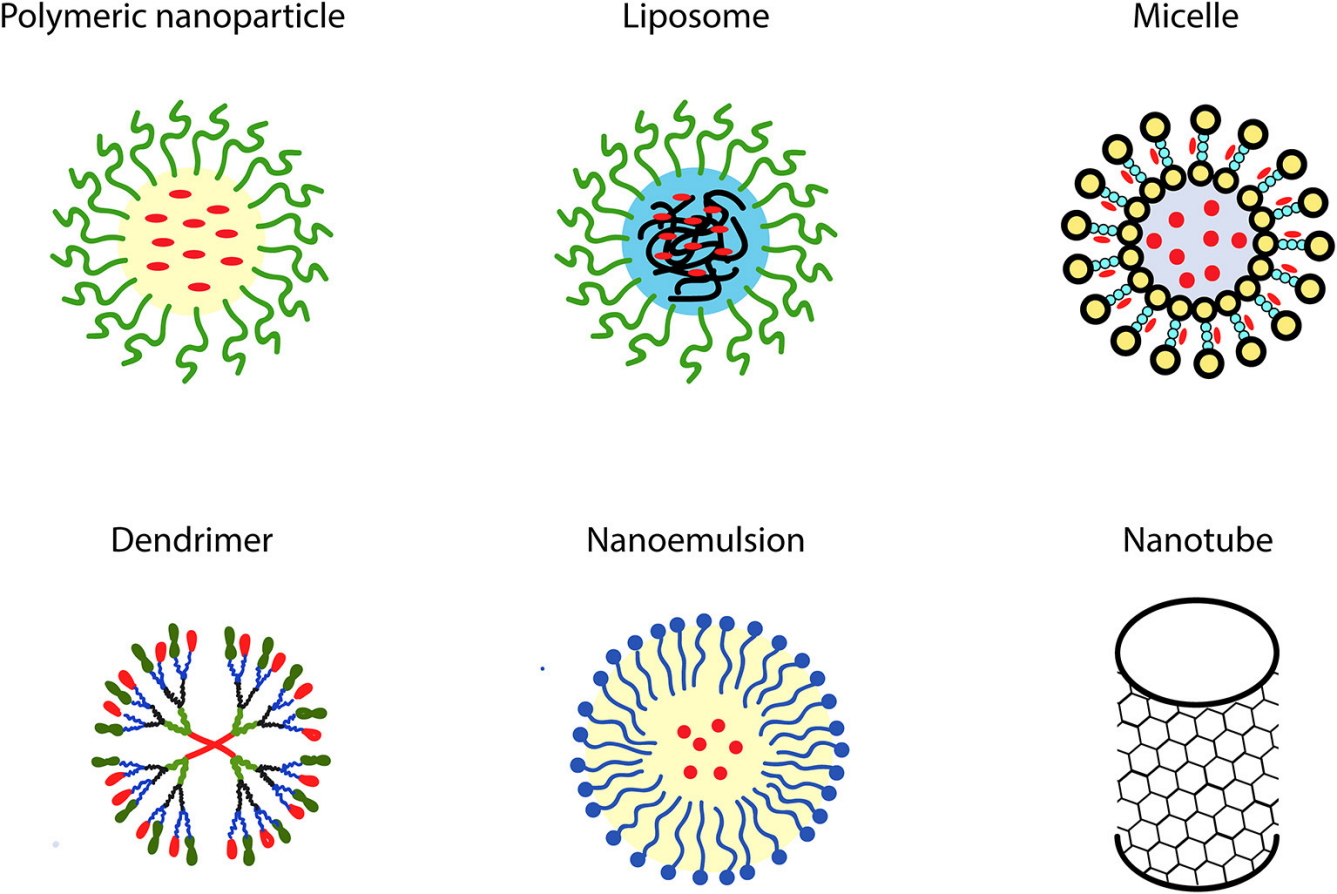
Chemical structures of the most commonly used phytoantioxidants.

Disappointing outcomes of clinical trials with synthetic antioxidants have resulted in doubts regarding the appropriateness of their use to combat atherosclerosis and other ROS-mediated degenerative diseases (Toledo-Ibelles and Mas-Oliva, 2018). Therefore, dietary supplementation with natural antioxidants derived predominantly from plant sources, such as polyphenols and carotenoids, has been proposed as a reasonable alternative to synthetic antioxidant intake (Xu et al., 2017; Serino and Salazar, 2018; Forni et al., 2019; Neha et al., 2019). Phytochemicals are secondary metabolites produced by plants to protect them from environmental stresses such as microbial infections, environmental pollutant exposures, temperature changes, and drought (Leonov et al., 2015). Due to such properties, they are considered to be promising candidates for developing healthspan- and lifespan-promoting interventions (Leonov et al., 2015). The efficiency of phytochemicals such as resveratrol, curcumin, catechins, genistein, and quercetin in counteracting various pathological conditions mediated by aging-related oxidative stress and associated chronic inflammation has been repeatedly reported (Corrêa et al., 2018; Martel et al., 2019). Chemical structures of the most commonly used phytotherapeutic compounds with potent antioxidant properties are presented in Figure 2 below.

One important problem is, however, that therapeutic potential of orally administered phytochemicals is substantially restricted because of their low bioavailability than is primarily attributed to their poor water solubility and intestinal permeability (Aqil et al., 2013; Khadka et al., 2014). In particular, the oral bioavailability was found to be about 1–2% for all quercetin (Kawabata et al., 2015; Li et al., 2016), resveratrol (Walle, 2011) and curcumin (Asai and Miyazawa, 2000; Yang et al., 2007), while for the epigallocatechin-3-gallate (EGCG) it is estimated to be about 0.1–0.3% only (Pervin et al., 2019). Therefore, novel biotechnological approaches are being actively developed by now to enhance the oral bioavailability and bioactivity of these substances. Recently, innovative nanotechnological applications have been developed to overcome this issue by improving the bioactivity of phytochemicals following oral administration.

### Therapeutic Advantages of Nanodelivery Systems

Among the most important features of orally administered nanomedicines, it is their capability to overcome the chemical and physical barriers in the gut such as the acidic pH of the stomach, intestinal mucosal lining and selectively permeable membranes of enterocytes (Moss et al., 2018). Physico-chemical characteristics of nanoparticles such as their metabolism, absorption, distribution and excretion depend on their size, hydrophobicity and charge. Size is an important parameter of nanoparticle that determines its pharmacokinetics, entry into the cell, and interaction with the immune system (Hoshyar et al., 2016). Surface properties of nanoparticles determine their hydrophilicity or hydrophobicity and also various biological responses, including cellular uptake, interactions with plasma proteins, particle removal as well as immune responses (Ajdary et al., 2018). Surface charge is also among the most important properties of nanoparticle substantially determining its cellular absorption and cytotoxicity (Fröhlich, 2012). Due to their properties, nanoparticles may protect loaded bioactive substances from degradation throughout the gastrointestinal digestion and cellular metabolism. Nanocarried bioactive agents are ultimately released in the gastrointestinal tract, in the circulatory system or in various tissues (Martínez-Ballesta et al., 2018). Their subsequent biological fate depends on their own chemical and physical properties and also on the site of release. Importantly, the location of bioactive release can be driven by using nanomaterials with certain surface chemistry; it makes possible the release of such therapeutics in particular tissues and body sites (Patra et al., 2018).

The nanostructures can act by either active or passive therapeutic targeting (Kydd et al., 2017). Following the passive nanodelivery mode, the loaded therapeutic agent is released by the erosion or diffusion of the delivering nanovector. The active delivery mode allows the controlled release of transported biomolecules at the targeted body sites. In this delivery mode, certain RNAs, proteins, lipids, carbohydrates, and small metabolite molecules are used as biomarkers to reach particular target sites (Conte et al., 2017). Selective targeting to specific tissues or body sites also becomes possible in this therapeutic modality by incorporating specific stimuli-responsive components which can be triggered by particular stimuli such as electric or magnetic field, light, pH, heating, ultrasound, and also by contact with concentrated ionic solutions or certain enzymes (Gu et al., 2018). Moreover, there exists a possibility to modify the physical surface properties of metallic nanoparticles, such as silver, gold, and iron oxide nanoparticles, so that they act as drug carriers by the active delivery mode (Kong et al., 2017). The use of organic nanocarriers is, however, considered to be more preferred because of their physico-chemical properties can be more finely tuned by modifying their chemical composition, shape, size, structural morphology, and characteristics of surface (Conte et al., 2017). The efficiency of nanodelivery of natural therapeutic agents is also dependent on their molecular weight. Increase of the molecular weight generally results in a decrease in efficiency of delivery of loaded compounds resulting in their lower bioavailability (Ganesan et al., 2018b). Different therapeutic nanodelivery systems can provide various health benefits depending on their properties, the features of loaded agents and also on desired therapeutic applications (Gupta and Xie, 2018; Rizvi and Saleh, 2018). In particular, natural compound-loaded nanoparticles provide many benefits over conventional formulations in terms of therapeutic potential. These benefits include improved epithelium permeability, enhanced stability, extended half-life, increased solubility and bioavailability, improved tissue targeting as well as minimized side effects (Date et al., 2016; Kermanizadeh et al., 2018). The nanotechnology-based systems are increasingly applied now to prevent and cure aging-associated pathological conditions, including neurodegenerative disorders such as Alzheimer's and Parkinson's diseases (Poovaiah et al., 2018; Ramanathan et al., 2018), cardiovascular diseases (Li et al., 2018; Taneja et al., 2019), obesity and type 2 diabetes (Bahadori et al., 2019; Tsou et al., 2019), and also tumors (Qiao et al., 2019).

### Therapeutic Nanodelivery Systems

The oral administration route is commonly considered as the most accepted way for drug delivery due to simplicity of administration, pain avoidance, patient compliance, and easy self-administration (Anselmo and Mitragotri, 2014). However, since the efficiency of oral drug delivery may be substantially reduced due to chemical and enzymatic barriers and also poor solubility in the gastrointestinal tract, the oral delivery of therapeutic compounds loaded into nanocarriers has become a subject of comprehensive investigation over the last years (Kermanizadeh et al., 2018). Delivery by nanocarriers generally may have varied therapeutic advantages. These advantages include, among others, an increased half-life, extended circulation time, enhanced mean residence time, and improved pharmacokinetic clearance of these agents from the body (Ravindran et al., 2018). Currently, many nanosized delivery systems intended for oral administration of phytotherapeutics have reached the clinical trial stage and are increasingly applied in clinical practice (Hajialyani et al., 2018). One important challenge in this research is developing novel multifunctional nanomaterials possessing properties allowing them to transfer particular therapeutics across various biological barriers and able to target specific cell types, tissues and organs in the body. Successful nanodelivery systems are characterized by optimal features for loading and release of therapeutic agent, long storage life, as well as high therapeutic efficacy with no or minimized side effects (Bilia et al., 2017; Piazzini et al., 2018). Among these nanodelivery systems, there are both solid (nanocrystals, lipid and polymeric nanoparticles) and liquid (including nanoliposomes, nanoemulsions, and nanopolymersomes) ones (Borel and Sabliov, 2014; Ganesan et al., 2018a). These systems are described in details in the subsequent sections.

### Nanoemulsions

Nanoemulsions include mixtures of immiscible liquids, such as e.g., water and oil (Jaiswal et al., 2015). Such nanosystems are generally prepared by either chemical or mechanical methods. Chemical methods result in spontaneous formation of emulsion droplets due to hydrophobic effects of lipophilic molecules which take place in the presence of emulsifiers. Mechanical methods include high-energy processes by which large emulsion droplets may be broken down into the smaller ones by various mechanical operations. The basic difference among nanoemulsions and conventional emulsions lies in the shapes and sizes of particles dispersed in a suspension. The droplet sizes in nanoemulsions ordinarily fall in the range of 20–200 nm.

### Nanoliposomes

Nanoliposomes represent nanosized self-assembled vesicles which consist of phospholipid bilayers entrapping one or more aquatic compartments (Chan and Král, 2018). There are fine oil-in-water (o/w) dispersions with droplet sizes ranging between 50 and 450 nm (Bozzuto and Molinari, 2015). Such nanodelivery systems may be produced by layer-by-layer electrostatic deposition technique. In this technique, charged polymers are added to a solution containing a charged template structure (Chun et al., 2013). Such template structures may be, e.g., emulsion droplets stabilized by ionic emulsifiers, liposomes composed of charged polar lipids, or hydrogel particles composed of charged biopolymers. The other method to produce nanoliposomes is the gentle hydration (the process of hydration of dried lipid films with an aqueous solution).

### Nanopolymersomes

Nanopolymersomes (NPS) are artificial vesicles with sizes from tens of nm up to 1 μm which enclose aqueous cavities, resulting from self-assembly of amphiphilic copolymers (Zhang and Zhang, 2017). The tunable properties of NPS allow to adjust them for different biomedical applications, e.g. as drug delivery vehicles or as artificial organelles (Pippa et al., 2016; Tuguntaev et al., 2016). They are synthesized by methods similar to those applied to producing polymeric nanoparticles (see below). The overall properties of NPS, including drug encapsulation and release capability, may be effectively tuned by using different biodegradable and/or stimuli-responsive block copolymers (Zhang and Zhang, 2017). Owing to their tunable properties, NPS are capable to encapsulate hydrophobic and hydrophilic molecules either in a membrane bilayer or in an aqueous core, respectively. Their advantages, in comparison with nanolipid carriers, include enhanced stability and versatility, and also controlled release (Rastogi et al., 2009). Due to these properties, NPS are considered to be potentially attractive drug carriers in many clinical applications.

### Nanocrystals

Nanocrystals are sub-micron (usually from 10 to 800 nm) colloidal dispersion systems consisting of pure (carrier-free) drug nanoparticles (Gigliobianco et al., 2018). They can be produced with either mechanical or chemical methods. The basic advantage of such nanosystems is reducing the particle size to nanoscale range, resulting in an increase of the particle surface area which is in contact with the dissolution medium (Singh and Lillard, 2009). Therefore, nanocrystal formulations are believed to have potential therapeutic benefits compared to the conventionally used pharmaceutical formulations. Among others, these benefits include improved saturation solubility and dissolution rate, and also high drug loading (Zhou et al., 2017).

### Solid Lipid Nanoparticles

Solid lipid nanoparticles (SLP) are similar to nanoemulsions, except that they contain lipids in solid phase. These are sub-micron colloidal nanocarriers ranging from 50 to 1,000 nm consisting of physiological lipids dispersed either in water or in aqueous surfactant solutions (Mishra et al., 2018; da Silva Santos et al., 2019). SLP are produced by high-energy methods such as microfluidization (high-pressure homogenization) and ultrasonication (a process that uses high-frequency sound energy to break apart particle agglomerates by expansion, cavitation and implosion of bubbles) (Mehnert and Mäder, 2001). The advantages of such nanoparticles include small size, large surface area, high drug loading efficiency, and the interaction of phases at the interface (Mishra et al., 2018). This type of nanocarriers was developed to overcome limitations of colloidal nanocarriers such as emulsions, liposomes, and polymeric nanoparticles since they can provide benefits such as targeted drug delivery and a good release profile (Naseri et al., 2015). The loading of therapeutic agents in such nanoparticles may occur in two ways: they can be either integrated in the core matrix or attached on the surface of nanoparticle (Lin et al., 2017). One important advantage of solid lipid nanoparticles is that they provide an option to entrap lipophilic molecules in stable particles without applying organic solvents. Such nanoparticles provide multiple therapeutic advantages due to their unique size-dependent properties and capacity to incorporate drugs. These advantages include feasibility for large-scale production, possibility of incorporating both lipophilic and hydrophilic drugs, high bioavailability of loaded drugs and low toxicity (Bayón-Cordero et al., 2019).

### Polymeric Nanoparticles

Polymeric nanoparticles include solid colloidal nanoparticles sized from 10 to 1,000 nm consisting of either synthetic or natural polymers (Crucho and Barros, 2017; Khan et al., 2017). These nanostructures are believed to have improved performance in energy storage compared to the bulk conducting polymers due to their high electrical conductivity, high electrochemical activity, large surface area, and short path lengths for the transport of ions (Pan et al., 2010). The methods for preparing polymer nanoparticles include nanoprecipitation, solvent evaporation, salting-out, dialysis, and supercritical fluid technology. They may also be directly synthesized by the polymerization of monomers by using techniques such as mini- and micro-emulsion, interfacial polymerization, and controlled/“living” polymerization methods (Krishnamoorthy and Mahalingam, 2015; Heinz et al., 2017). Surface modification of the polymeric nanoparticles is achieved via self-assembly of block copolymers containing segments that form the core of the polymer nanoparticle and segments that form the outer surfactant shell upon assembly (Heinz et al., 2017). Drug-loaded polymer nanoparticle synthesis is carried out using biodegradable and biocompatible polymers or copolymers, in which therapeutic agents may be encapsulated or entrapped within the carriers or be physically adsorbed on or chemically linked to the nanoparticle surfaces. Polymeric nanoparticles generally fall into two major subtypes, i.e., nanospheres where loaded agents are uniformly dispersed and nanocapsules where therapeuticals are confined to the inner aqueous or oily cavities surrounded by polymeric membranes (Grottkau et al., 2013). The attractive capabilities of these nanostructures include water solubility, small size, storage stability, biodegradability, long shelf life, and non-toxicity (Kamaly et al., 2016). Owing to their physico-chemical characteristics, polymeric nanoparticles also demonstrate good encapsulation efficiency, and also improved solubility and stability of hydrophobic drugs. These properties allow to minimize toxicity of loaded drugs, permitting a controlled release at targeted sites of the body at relatively low doses (Kamaly et al., 2016).

**Figure 3:**
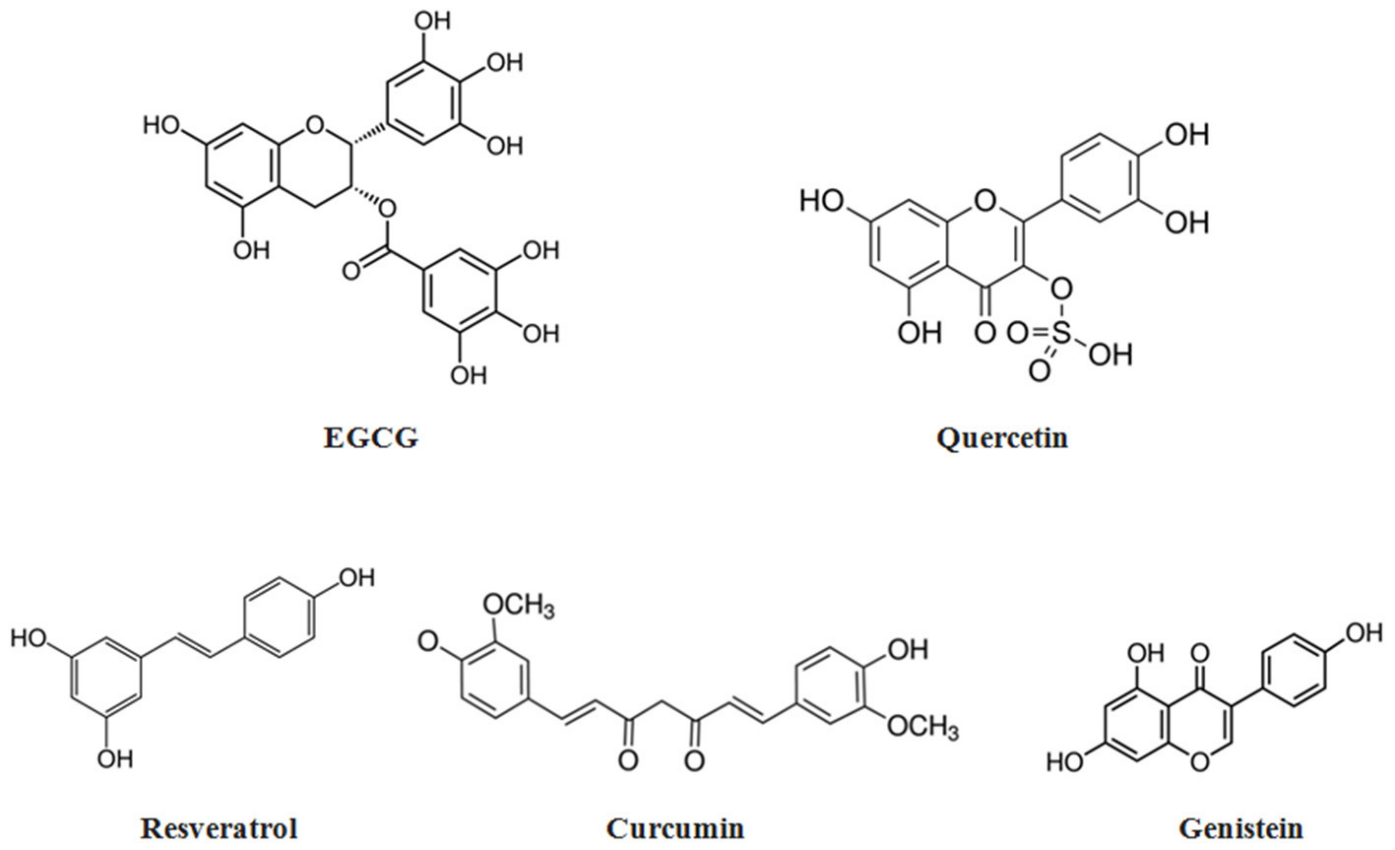
Graphical representations of the most common types of nanocomposites.

Some of the most widely used solid and liquid nanodelivery systems are schematically presented in Figure 3.

### Metallic Nanoparticles

Metallic nanoparticles with diameters ranging from 1 to 100 nm such as silver, gold, copper, magnesium, aluminum, titanium, and zinc ones are increasingly being applied for either passive or active drug delivery in different biomedical applications. An important point is that metallic nanoparticles can be synthesized and modified with various chemical functional groups which allow them to be conjugated with different drugs of interest to target certain cells and tissues (Mody et al., 2010). Their obvious advantages in clinical applications include relatively simple synthesis, easy chemical modification, biocompatibility, and tunable biophysical properties (Lushchak et al., 2018).

### Nano-Antioxidants

One of major factors limiting clinical use of most nanoparticle formulations is their capability to induce oxidative stress at cellular level (Fu et al., 2014; Zuberek and Grzelak, 2018). One important point in this regard, however, is that levels of generated ROS are dependent on the nanoparticle concentration to which the cell is exposed. Different nanomaterials may influence cellular redox environment by either stimulating or inhibiting ROS production, and a biphasic dose-response relationship characterized by a low-dose stimulation and a high-dose inhibition (hormetic response) could likely play a crucial role in redox-modulating effects induced by nanoparticles (Iavicoli et al., 2018). The exposure to high nanoparticle concentrations usually results in excess ROS generation and overloading of endogenous antioxidant systems, eventually causing cytotoxicity, and inflammation (Nel et al., 2006). Determination of maximal admissible doses is considered therefore to be critical to avoid adverse health outcomes. Accumulating evidence, however, suggests that low-level nanoparticle exposures may unexpectedly enhance antioxidant defense ability and depress oxidative stress (Abdal Dayem et al., 2017). Some nanomaterials have been found to be able to exhibit enzyme-like antioxidant properties by being capable to scavenge ROS and other free radicals, thereby reducing oxidative injury (Lushchak et al., 2018). Such nanomaterials are generally referred to as “nano-antioxidants” (Watal et al., 2013; Du et al., 2014; Sandhir et al., 2015). Nano-antioxidants include non-organic nanoparticles such as metallic nanoparticles with intrinsic antioxidant properties (Lushchak et al., 2018) as well as nanoparticles functionalized with antioxidant enzymes or natural antioxidants (Sandhir et al., 2015). The antioxidant potential of nanocarriers loaded with phytotherapeutic agents is described in more detail in the following subsections.

### Nano-Phytoantioxidants: New Promise in Anti-aging Research

Many nanodelivery systems loaded with plant-based bioactive compounds have been demonstrated to be efficacious in modulating oxidative stress and related chronic inflammation which mediates most aging-associated disorders. Results from studies reporting antioxidant effects of such nanodelivery systems are discussed in subsections below.

### Nano-Resveratrol

Resveratrol (3,5,4′-trans-trihydroxystilbene) is a polyphenol compound found in grapes skin and seeds, and, in lesser amounts, in several other plant sources (Salehi et al., 2018). In plants, it acts as a phytoalexin, protecting them from pathogens such as fungi and bacteria. In a number of animal models, potent antioxidant properties of resveratrol have been consistently reported. The antioxidant capacity of this polyphenolic compound strongly depends on the redox properties of phenolic hydroxyl groups and the potential for electron delocalization across its chemical structure (Sedlak et al., 2018). Resveratrol has three hydroxyl groups (see Figure 2 for illustration) known to play crucial roles in ROS scavenging. Moreover, these hydroxyl groups help to chelate metal ions, which is an essential feature in the prevention of ROS production (Gülçin, 2010). Furthermore, the cellular defense might be achieved by ability of resveratrol to act not only as a direct antioxidant, but also as an indirect manner, as an endogenous antioxidant system inducer (Salehi et al., 2018). Resveratrol can trigger Nrf2 transcription factor known to regulate variety of antioxidant enzymes (Lushchak, 2011; Smith et al., 2016). In addition, the antioxidant properties of this polyphenolic compound might be attributable to its effects as a gene regulator. Particularly, it was shown that down-regulating the expression of NADPH oxidase may inhibit ROS production (Xia et al., 2017). Resveratrol can also reduce mitochondrial superoxide production by stimulating mitochondrial biogenesis, prevent superoxide generation from uncoupled endothelial nitric oxide synthase by up-regulating the tetrahydrobiopterin-synthesizing enzyme GTP cyclohydrolase I, and also increase the expression of different antioxidant enzymes (Xia et al., 2017).

Antioxidant properties of resveratrol are believed to be responsible for most health-promoting effects of this substance (Carrizzo et al., 2013). Moreover, its anti-inflammatory, cardioprotective, neuroprotective, and also anti-cancer properties have also been repeatedly reported. Therefore, it is regarded as one of the most promising anti-aging natural compounds now (Wahl et al., 2018). Its anti-aging effects are believed to be attributed to capacity to activate mammalian silent information regulator 1 (SIRT1) and modulate activity of certain important regulatory proteins known to be crucially implicated in aging processes, including Akt/protein kinase B, peroxisome proliferator-activated receptor coactivator-1α (PGC-1α), NF-κβ and FoxO, and also cellular processes such as apoptosis, angiogenesis, mitochondrial biogenesis, lipid metabolism and gluconeogenesis (Camins et al., 2009; Pannu and Bhatnagar, 2019). Many of these activities are similar to those found in calorie restriction research, thereby demonstrating the potential of resveratrol as a calorie restriction mimetic (Li J. et al., 2017). Its effectiveness and safety have been well-documented in many animal models and in clinical trials. The therapeutic potential of resveratrol has been reported for various aging-associated pathological conditions such as metabolic syndrome, obesity, type 2 diabetes, cardiovascular disorders, hypertension, stroke, chronic kidney and inflammatory diseases, dementia and also breast, and colorectal cancers (Berman et al., 2017; Ramírez-Garza et al., 2018; Singh et al., 2019).

The therapeutic applicability of resveratrol is, however, substantially restricted through its extensive hepatic and presystemic metabolism (Pangeni et al., 2014; Smoliga and Blanchard, 2014). Moreover, the water solubility of this phytochemical is very low, thereby causing poor absorption by oral administration (Chauhan, 2015). Considering this, many preclinical and clinical trials are in progress now to create structurally modified derivatives of resveratrol having higher bioavailability upon ingestion (Popat et al., 2013). One example is a micronized liquid formulation of trans-resveratrol, SRT501, which demonstrated roughly five times higher bioavailability compared to non-micronized compound (Elliot and Jirousek, 2008). In several clinical trials, however, SRT501 was not well-tolerated and resulted in side effects, including vomiting and diarrhea, which caused dehydration and renal failure in some patients (Popat et al., 2013). It prompted the search for nanomaterials acting similarly to SRT501, but without its side effects (Smoliga et al., 2012). Recently, some such nanosized resveratrol-loaded formulations have been investigated for their potential clinical utility. The bioavailability of orally delivered trans-resveratrol loaded in lipid-core nanocapsules was found to be two times higher than that of the free trans-resveratrol in brain, kidney and liver of male Wistar rats (Frozza et al., 2010). Nanodelivery also resulted in improved gastrointestinal safety in this model. The bioavailability of the folate-conjugated human serum albumin-encapsulated resveratrol nanoparticles was also shown to be 6-fold higher compared to native resveratrol following the intravenous administration (Lian et al., 2019).

Several studies have demonstrated antioxidant properties of nano-encapsulated resveratrol. For instance, in the study by Chen et al. (2015), resveratrol nano-encapsulated in self-micro-emulsified drug delivery system exhibited higher antioxidant capability and reduced toxicity compared to free resveratrol. Resveratrol loaded in nanoliposome carriers (size from 103 to 134 nm) also exhibited more pronounced radical scavenging effect when compared to pure resveratrol (Vanaja et al., 2013). High ROS scavenging efficiency was also demonstrated for the vitamin E-loaded resveratrol nanoemulsion (an average globule diameter of about 100 nm) in patients with Parkinson's disease (Pangeni et al., 2014). The activities of endogenous antioxidant enzymes, including SOD, and GSH lelels were shown to be significantly higher, and levels of malondialdehyde was significantly lower in the resveratrol nanoemulsion-administered group. Resveratrol loaded in zein nanoparticles with bovine serum albumin-caffeic acid conjugate (particle size from 206 to 217 nm) was demonstrated to exert significantly higher cellular antioxidant activity than resveratrol alone (Fan et al., 2018a). Anti-inflammatory abilities of resveratrol-loaded nanoparticles, such as galactosylated poly(lactic-co-glycolic acid) nanoparticles, have been also reported (Siu et al., 2018).

### Nano-Curcumin

Curcumin [1,7-bis(4-hydroxy-3-methoxyphenyl)-1,6-heptadiene-3,5-dione] is a polyphenol extracted from rhizome of the turmeric plant, *Curcuma longa*. It is traditionally used in Asian countries as herbal treatment (Hewlings and Kalman, 2017). This compound has three chemical components in its structure, including one diketone moiety and two phenolic groups (Figure 2). The active functional groups of curcumin may undergo oxidation via electron transfer and hydrogen abstraction processes (Priyadarsini, 2014). In particular, the antioxidant activity of curcumin is determined by methylenic hydrogen and o-methoxy phenolic groups. Moreover, the β-diketone groups can chelate ions transition metals; some of these metal complexes exhibit antioxidant enzyme-mimetic activities (Priyadarsini, 2013).

Along with antioxidant properties, this polyphenol compound exhibit anti-inflammatory, anti-neurodegenerative and anti-cancer activities (Sarker and Franks, 2018). The potential of curcumin in preventing and treating various aging-associated pathological conditions has been repeatedly reported (Sundar et al., 2018). These conditions include oxidative stress, inflammation, atherosclerosis, cardiovascular and neurodegenerative diseases, type 2 diabetes, osteoporosis, rheumatoid arthritis, age-related kidney and ocular diseases, and also cancer. Over the last decade, the healthspan-promoting potential of this compound has been comprehensively investigated in clinical trials (Salehi et al., 2019). Its bioavailability is, however, limited through its low water solubility and gastrointestinal stability (Kumar et al., 2010). Innovative nanodelivery strategies are currently developed to overcome these limitations (Flora et al., 2013; Ahmad et al., 2014). Both *in vitro* and *in vivo* evidence suggests that nanocurcumin formulations have better healthspan-promoting properties than conventional (native) curcumin. The water solubility and bioavailability of this compound were shown to be significantly enhanced by nano-encapsulation with lipid or polymeric nanoparticles, nanogels and dendrimers, and also by conjugating to metal oxide nanoparticles (Shome et al., 2016). In particular, the oral bioavailability of poly lactic-co-glycolic acid (PLGA) curcumin nanoformulation has been shown to be 22-fold higher than that of native curcumin (Tsai et al., 2011). In a rat model of cerebral ischemia, curcumin-loaded solid lipid nanoparticles demonstrated a 16-fold higher bioavailability of the curcumin in the brain than that of free curcumin (Kakkar et al., 2013). Similarly, oral bioavailability and brain distribution of curcumin were shown to be significantly enhanced in N-trimethyl chitosan surface-modified solid lipid nanoparticles compared to those of native curcumin (Ramalingam and Ko, 2015).

In *in vitro* experiments with a Caco-2 cell line, evidence was also obtained that bovine serum albumin dextran nanoparticles (size up to 200 nm) loaded with curcumin may exert significant cellular antioxidant activity (Fan et al., 2018b). The addition of curcumin-loaded nanocapsules produced from the Eudragit L-100 polymer to the diets of dairy sheeps resulted in a higher antioxidant capacity and lower lipid peroxidation in their milk (Jaguezeski et al., 2019). Anti-inflammatory activities of different curcumin-loaded nanocomposites were also repeatedly reported (Wang et al., 2015a; Ameruoso et al., 2017; Dewangan et al., 2017; El-Naggar et al., 2019).

### Nano-Quercetin

Quercetin (3,5,7,3′,4′-pentahydroxyflavone) is a bioactive flavonoid with strong antioxidant properties, including its effects on ROS levels and also on various cellular signal transduction pathways and activities of antioxidant enzymes. In particular, it has displayed the capacity to prevent the oxidation of low-density lipoproteins by scavenging free radicals and chelating transition metal ions. The antioxidant activity of this polyphenolic flavonoid compound is considered to be mainly attributed to its metal ion complexes and complex ions (Xu et al., 2019). When quercetin reacts with a free radical, it donates a proton and becomes a radical itself. The resulting unpaired electron is, however, delocalized by resonance, thereby making the quercetin radical too low in energy to be chemically reactive (Flora, 2009). The antioxidant ability of quercetin is due to the presence of phenolic hydroxyl groups which are accessible to oxidizing agents (Figure 2). There are the B ring o-dihydroxyl groups, the 4-oxo groups in conjugation with the 2,3-alkene, and the 3- and 5-hydroxyl groups (Haq and AlAmro, 2019). All these functional groups may donate electrons to the rings, which increase the number of resonance forms available in addition to those created by the benzene structure. In addition to antioxidant properties, quercetin is known to demonstrate anti-inflammatory, anti-obesity, anti-diabetic, anti-atherosclerotic, anti-hypercholesterolemic, and anti-hypertensive activities (Anand David et al., 2016). However, its health benefits are limited due to low bioavailability (<2% of the administered dose) (Kawabata et al., 2015; Li et al., 2016). Over the last years, innovative nanotechnology-based approaches have been developed to enhance the quercetin bioavailability. Among them, quercetin-loaded solid lipid nanoparticles have been recently developed that exhibited a significantly improved bioavailability compared to pure quercetin powders (Vijayakumar et al., 2016).

Quercetin-loaded nanoparticles have been also shown to be able to improve antioxidant defense mechanisms in animal models. For example, in a streptozotocin-induced diabetic rat model, quercetin-loaded poly(lactic-co-glycolic acid) nanoparticles with size about 180 nm demonstrated antioxidant properties similar to those of free quercetin (Chitkara et al., 2012). The same doses of this nanoformulation used in the every-fifth-day dosing regimen have been found to be sufficient to bring effects similar to those from daily doses of the oral quercetin suspension. Similar effects were also observed in pancreas and kidneys for CAT and SOD activities. In rat models, self-emulsifying nanoformulation of quercetin also exhibited a significantly higher antioxidant potential compared to free quercetin when evaluated as a function of capability to combat doxorubicin- and cyclosporin A-induced cardiotoxicity and nephrotoxicity, respectively (Jain et al., 2013). Elevated SOD and CAT activites, GSH levels and amelioration of lipid peroxidation and protein carbonylation were also observed in alloxan-induced diabetic mice treated with quercetin-loaded nanorods (Alam et al., 2016). In addition, quercetin-loaded silica nanoparticles were found to be able to ameliorate inflammatory conditions in different cell lines (Lee et al., 2016).

### Nano-Genistein

Genistein [4′,5,7-trihydroxyisoflavone or 5,7-dihydroxy-3-(4-hydroxyphenyl) chromen-4-one] is a soy phytoestrogenic isoflavone possessing potent antioxidant activity (Spagnuolo et al., 2015). The antioxidant properties of this isoflavone were shown to be dependent, in particular, on its ability to induce the expression of genes encoding antioxidant enzymes including SOD and CAT (Park et al., 2010). It has been demonstrated to be efficient in combating many age-related disorders, including neurodegenerative diseases, osteoporosis, obesity, type 2 diabetes, and cancer (Saha et al., 2014; Ganai and Farooqi, 2015). However, the clinical use of this compound is often limited due to its low bioavailability. Moreover, it may provoke endocrine-disrupting and toxic effects, especially when applied in high doses (Patisaul, 2017). To overcome these potential side effects, innovative nanotechnological solutions have been recently proposed (Rassu et al., 2018). For example, enhanced oral bioavailability has been revealed in genistein-loaded polymeric nanomicelles in comparison with that for free genistein (Kwon et al., 2007). According to the authors, this effect could be due to higher solubility and gastrointestinal release of nanomicelle-loaded genistein. The oral bioavailability was also found to be improved in genistein loaded in solid lipid nanoparticles compared to that for its suspensions or bulk powders (Kim J. T. et al., 2017). Recently, Pool et al. (2018) found in studies with HT29 human colon cancer cells that incorporation of genistein into small (~33 nm) PEGylated silica nanoparticles may substantially potentiate its antioxidant effects. These effects were mediated via modulation of endogenous CAT and SOD activities, and H_2_O_2_ production. It leads to induction of apoptosis and autophagy, two processes related to cell death, while only apoptosis was activated by free genistein.

### Nano-Epigallocatechin-3-Gallate

Epigallocatechin-3-gallate [EGCG, (2R, 3R)-5,7-Dihydroxy-2-(3,4,5-trihydroxyphenyl) chroman-3-yl 3,4,5-trihydroxybenzoate] is one of the major polyphenol (catechin) found in green tea. The molecule of EGCG is very complex (Figure 2), it consists of a gallocatechol group and a gallate ester linked to the flavanol core structure (Botten et al., 2015). The gallocatechol rings in the EGCG structure determine its antioxidant properties since they are able to directly capture free radicals (Braicu et al., 2013). There is convincing evidence that this compound has a stronger antioxidant potential than other green tea catechins, and that it is even more effective in ROS scavenging than vitamins C and E (Rice-Evans et al., 1995). EGCG has been also repeatedly shown to exhibit anti-inflammatory, anti-atherogenic and anti-cancer activities (Singh et al., 2011; Shi et al., 2018). The data from epidemiological studies devoted to investigating EGCG are, however, controversial and often conflicting with *in vitro* findings. This ambiguity could be attributed to its poor stability and bioavailability (Mereles and Hunstein, 2011; Krupkova et al., 2016; Chu et al., 2017). Therefore, in attempt to improve the bioavailability of EGCG, innovative nanodelivery systems have been extensively used (Granja et al., 2017). In particular, larger stability and enhanced potential for oral delivery were found in EGCG-loaded solid lipid nanoparticles than those for non-processed EGCG (Frias et al., 2016). Two-fold higher bioavailability of pH-sensitive EGCG-loaded polymeric nanoparticles compared to the native EGCG powder was also demonstrated (Zhang and Zhang, 2018). EGCG-loaded nanoparticles have been also shown to exert antioxidant properties in *in vitro* models. One example is the study by Avadhani et al. (2017), who synthesized nanotransfersomes containing EGCG and hyaluronic acid (vesicle size ~100 nm) aiming to synergize UV radiation-protective capabilities of these compounds. This optimized nanotransfersomal formulation was found to be able to reduce the lipid peroxidation and intracellular ROS levels, and also to increase the viability of human keratinocytes *in vitro*. EGCG-loaded nanoparticles also have been shown to demonstrate anti-inflammatory activities in *in vitro* models (Wu et al., 2017).

### Nano-Phytoantioxidants in Therapy of Aging Disorders: Preclinical Evidence

Accumulating evidence indicates that nano-phytoantioxidants have a potential in preventing and treating a wide range of aging-associated pathological conditions. In several animal models, orally administered nano-phytoantioxidants demonstrated a more powerful potential in combating cardio-metabolic disorders than that of their raw forms. Substantial anti-diabetic potential was, e.g., shown in db/db diabetic mice administered with nanoparticles loaded with the isoquinoline alkaloid berberine (Xue et al., 2013, 2015). These mice demonstrated a substantial suppression of body weight gain as well as improved glucose tolerance and insulin sensitivity. Substantial hypoglycemic effect was also observed in both normal and diabetic rats administered with selenium-layered nanoparticles loaded with extracts of mulberry leaf and *Pueraria lobata* (Deng et al., 2019). These nanoparticles were also able to attenuate the oxidative damage, promote glucose utilization by adipocytes and enhance pancreatic function. Solid lipid nanoparticles loaded with the bioactive constituent of kudzu roots, puerarin, showed three times higher bioavailability following oral administration in heart and brain compared to free puerarin (Luo et al., 2011). Treatment with curcumin-loaded nanoparticles led to attenuation of palmitate-induced cardiomyocyte apoptosis in H9C2 embryonic rat heart-derived cells (Li J. et al., 2017). Use of colloidal curcumin nanoparticles dissolved in gum ghatti solution resulted in the restoration of the left ventricular fractional shortening (a violation arising from heart failure following myocardial infarction) in male rats (Sunagawa et al., 2012). In rats administered with solid lipid nanoparticles loaded with extract from *Dracocephalum moldavica L*., more pronounced therapeutic effects against myocardial ischemia-reperfusion injuries have been revealed in comparison with that of the non-modified extract (Tan et al., 2017). Evidence was also obtained that nano-phytoantioxidant therapy can be a promising solution in treating rheumatoid arthritis, a systemic autoimmune disease caused by chronic inflammatory process occurring in consequence of age-related decline of immune function (immunosenescence) (van Onna and Boonen, 2016). In an adjuvant-induced arthritis rat model, either oral or topical administration of piperine-loaded solid lipid nanoparticles caused substantial reduction of the pro-inflammatory cytokine tumor necrosis factor alpha (TNFα) levels, assuming anti-rheumatic therapeutic potential of such a treatment (Bhalekar et al., 2017). In the same model, the protective potential of curcumin-loaded solid lipid nanoparticles in ameliorating adjuvant-triggered arthritis through attenuating oxido-inflammatory and immunomodulatory cascades was demonstrated (Arora et al., 2015). Nano-phytoantioxidants have been also shown to be efficient for improvement in bone biomechanical parameters and biochemical markers during osteoporosis, a chronic disease characterized by an age-associated deterioration in bone mass and micro-architecture (Barry et al., 2016). Curcumin-loaded poly(lactic-co-glycolicacid) nanoparticles have been found to potentiate protective effects of curcumin against the bone loss in ovariectomized rats (Ahn et al., 2017). In the same model, quercetin-based solid lipid nanoparticles were more effective in restoring bone mineral density in osteopenic animals than free quercetin (Ahmad et al., 2016). In an ovariectomized C57Bl/6 mice model, the administration of gold curcumin-loaded β-cyclodextrin conjugated nanoparticles also led to improved bone density and prevented bone loss (Heo et al., 2014).

Innovative nanobiotechnology-based approaches have been also recently developed in anti-cancer therapy. These approaches, in particular, allow drug delivery directly to tumor sites without damaging nearby healthy tissues (Ahmad et al., 2018). In *in vitro* studies, enhanced anti-tumor activity was obtained, compared to respective unmodified substances, for nanoengineered phytobioactive compounds exerting antioxidant and anti-inflammatory activities such as resveratrol (Rodenak-Kladniew et al., 2017; Wang et al., 2017), curcumin (Meena et al., 2017; Dhivya et al., 2018; Montalban et al., 2018; Wang W. et al., 2018; Ni et al., 2019; Somu and Paul, 2019; van der Vlies et al., 2019), EGCG (Chavva et al., 2019), berberine (Wang et al., 2014a; Zheng et al., 2018), aloe-emodin (Wang et al., 2012; Chen et al., 2014), and oridonin (Wang et al., 2014b). In mouse models of the induced cancer, substantial inhibition of tumor proliferation and angiogenesis and also enhanced levels of apoptosis of cancerous cells have been found in animals administered, either orally or intravenously, with nanoparticles co-loaded with curcumin, along, or in combination with particular anti-cancer drugs (Wang et al., 2015b, 2016; Yang et al., 2015; Yan et al., 2016; Cui et al., 2017; Kumari et al., 2017; Li C. et al., 2017), in comparison to those for free substances. Substantial anti-tumor properties have been also shown for nanoparticles loaded with resveratrol (Xu et al., 2016; Zhang et al., 2019), EGCG (Siddiqui et al., 2014; Tang et al., 2018), quercetin (Gao et al., 2012; Zhu et al., 2017), and berberine (Wang Y. et al., 2018).

**Figure 4:**
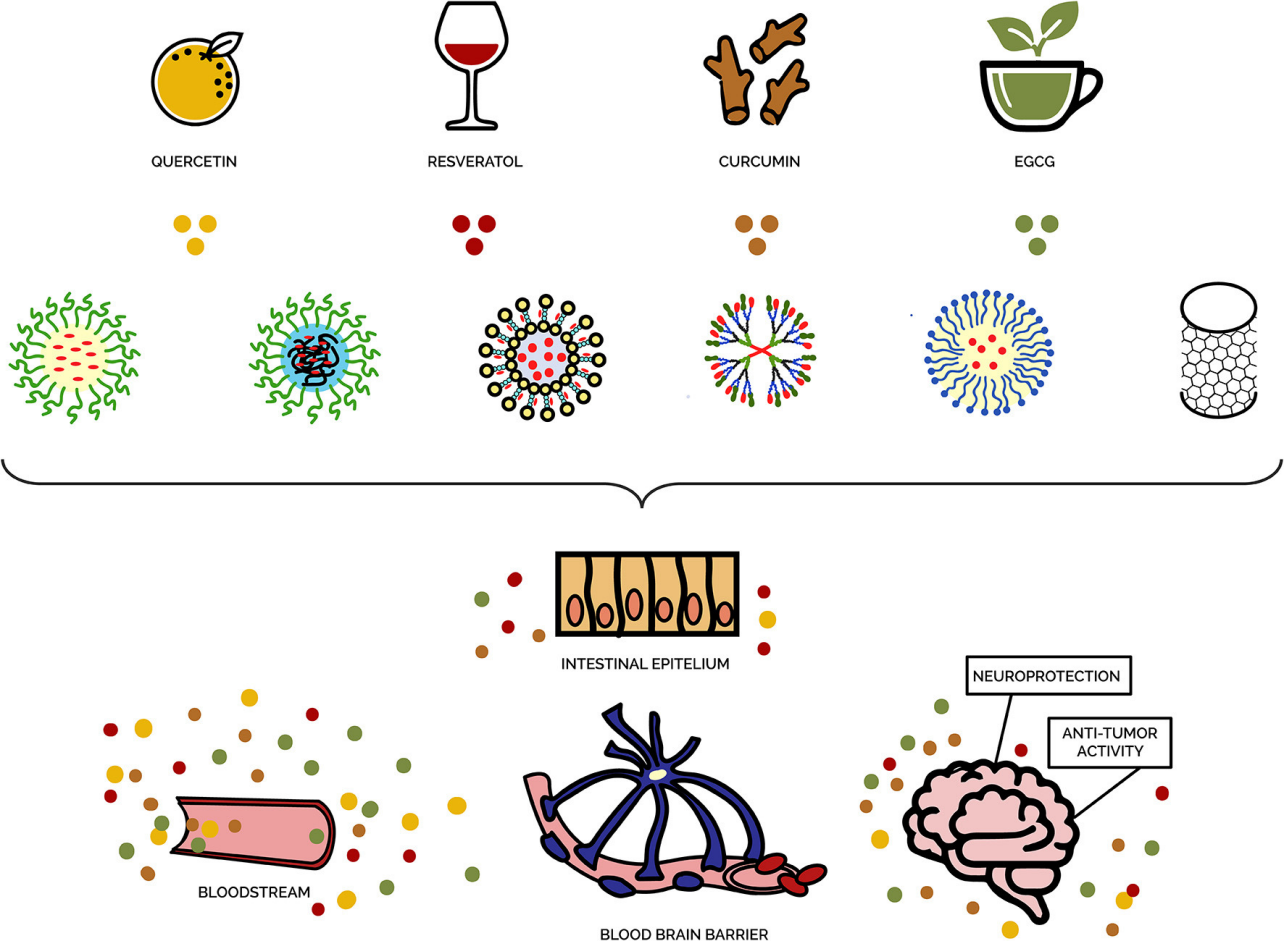
Schematic representation of nanotechnology-based systems used for brain delivery of phytoantioxidant-loaded nanodelivery systems.

The effectiveness of nanotechnology-based systems was also shown in combating neurodegenerative disorders such as Alzheimer's and Parkinson's diseases (Teleanu et al., 2018; Saeedi et al., 2019). Developing such approaches seems to be especially important for this therapeutic area since treating these disorders is a very difficult task because of the existence of the blood-brain barrier representing the most important obstacle in delivering pharmaceuticals to the brain. Tunable biophysical properties of nanocomposites allowing to overcome blood-brain barrier make them, potentially, highly useful in these therapeutic applications (Henrich-Noack et al., 2019; Sharma et al., 2019; see also Figure 4 for schematic representation).

In particular, increased oral bioavailability to the brain of nanotechnology-based delivery systems such as curcumin-loaded solid lipid nanoparticles (Ramalingam and Ko, 2015, 2016) and resveratrol-loaded N-trimethyl chitosan-g-palmitic acid surface-modified solid lipid nanoparticles (Ramalingam et al., 2016) has been demonstrated compared to the native forms of these phytobioactive compounds. Various nanocomposites administered by intranasal or intravenous routes also provided improved bioavailability to the brain in comparison with that for free drug administration (Gao, 2016). For example, quercetin-loaded solid lipid nanoparticles provided enhanced quercetin delivery to the brain along with improved antioxidant effect to brain cells compared to those of pure quercetin, as well as improved memory retention in a rat model of Alzheimer's disease (Dhawan et al., 2011). Increased bioavailability in brain cells was also observed for nanoparticles loaded with piperine (an active ingredient of black pepper) (Yusuf et al., 2012). In an animal model of Alzheimer's disease, the piperine-loaded nanoparticles exhibited therapeutic effects on disease progression, supposedly by reducing oxidative stress and cholinergic degradation. The enhanced bioavailability and improved treatment efficiency of nanoparticles loaded with curcumin, such as curcumin-loaded solid lipid nanoparticles (Kakkar and Kaur, 2011) and poly (lactic-co-glycolic acid) nanoparticles (Cheng et al., 2013) were also shown in mouse models of Alzheimer's disease. Resveratrol and grape extract-loaded solid lipid nanoparticles were also reported to have therapeutic potential for the treatment of this disease (Loureiro et al., 2017). The promising therapeutic potential in preventing and treating Alzheimer's disease has been found for quercetin-loaded nanoparticles as well (Han et al., 2018). The authors assumed that this potential was likely driven by the activity of these nanoparticles to inhibit amyloid β aggregation and scavenge free radicals. In a rat model of Alzheimer's disease, evidence was obtained that aluminum chloride-induced adverse neurobehavioral impairments may be attenuated by EGCG-loaded nanoparticles via reducing the formation of neurofibrillary tangles and neuritic plaques (Singh et al., 2018). Moreover, in a mouse model of familial Alzheimer's disease, oral administration with nanoparticles loaded with ascorbic acid and EGCG was shown to enhance accumulation of EGCG in the brain, as well as to increase the number of synapses and reduce amyloid-beta plaque/peptide accumulation and neuroinflammation (Cano et al., 2019). Nanobiotechnology-based approaches were shown to have a therapeutic potential in treating Parkinson's disease as well. For example, resveratrol-loaded polysorbate 80-coated poly (lactide) nanoparticles showed protective neuroprotective effects against neurochemical and behavioral impairments caused by neurotoxin (1-methyl-4-phenyl-1,2,3,6-tetrahydropyridine) known to damage dopaminergic neurons and induce Parkinson-like symptoms in a mouse model of Parkinson's disease (da Rocha Lindner et al., 2015).

## Conclusions

Oxidative stress caused by imbalance between ROS production and scavenging is undoubtedly one of key contributing factors in most aging-associated pathological conditions. Therefore, the development of therapeutic modalities to combat adverse consequences of oxidative stress, including damage to vital biomolecules, accelerated telomere attrition and systemic inflammation, is one of the most important tasks in current geroscience research. For several decades, dietary supplementation with chemically synthesized antioxidants has been considered as the most appropriate way to prevent and treat ROS-linked disorders. However, more recent studies provided rather controversial and often discouraging results, including increased mortality in those persons who regularly consume synthetic antioxidants such as β-carotene, vitamin A, and vitamin E (Bjelakovic et al., 2014). Such discouraging results can most likely be explained by the fact that, while assumption on pro-health potential of antioxidants is mostly based on *in vitro* assays, these assays may not reflect the physiological mechanisms operating *in vivo* (Shen et al., 2007; Ndhlala et al., 2010). Moreover, the ambiguity of results obtained from epidemiological studies could be likely explained by dichotomous roles of antioxidants in ROS production (Halliwell, 2000; Milisav et al., 2018) and also by important roles of ROS in different vital processes (Schieber and Chandel, 2014; Bardaweel et al., 2018; Milkovic et al., 2019). In addition, as oxidative stress and chronic inflammation coexist in aging-associated pathological conditions, the failing of clinical trials with synthetic antioxidants may be explained by inability to simultaneously target both oxidative stress and inflammatory responses (Biswas, 2016). Indeed, such pharmaceuticals may block several pro-oxidative and/or pro-inflammatory pathways but at the same time strengthen others. On the basis of these considerations, dietary supplementation with natural antioxidants (mostly polyphenols) seems to be a reasonable alternative to synthetic antioxidant intake since natural phyto-antioxidants are known to be able to effectively counteract both oxidative stress and inflammation (Petersen and Smith, 2016; Ganesan et al., 2017). The only problem is that *in vivo* activities of natural antioxidants may be limited owing to their low bioavailability (Shen et al., 2007). Therefore, effects obtained in *in vitro* experiments could likely be caused by much higher doses than those normally contained in human diet (Scalbert et al., 2005). Therefore, development of nanotechnology-based applications for targeted delivery of bioactive phenolic compounds with antioxidant properties to treat age-related chronic diseases is an urgent task of biotechnological research.

The use of nanocarrier-based systems was shown to enhance the solubility and stability of delivered bioactive phytochemicals, increase their gastrointestinal absorption, and protection from premature enzymatic degradation (Conte et al., 2017; Martínez-Ballesta et al., 2018). Conclusive evidence suggests that bioavailability of nanoparticle-encapsulated phytochemicals can be 5–10 times higher than that of native formulations (Ganesan et al., 2018a,b). Moreover, such mode of oral delivery may also result in prolonging the circulation time of these compounds, thereby reducing their potential toxicity and side effects. The implementation of nanotherapeutic approaches would likely provide an opportunity to overcome many shortcomings of conventional therapeutic strategies. Using phytochemical-loaded nanoformulations is regarded by many authors as a clinical equivalent to standard treatments with synthetic antioxidants, but with minimizing side effects (Anand et al., 2017). In addition, the beneficial feature of nanodelivery approach is that it can provide an opportunity to deliver phytochemicals to certain organs, such as the brain, following the oral administration.

In conclusion, despite delivery of phyto-antioxidants by nanodelivery systems has apparent healthspan-promoting potential, several important challenges still remain to be addressed. Nanocarriers used to encapsulate phytotherapeuticals have to be comprehensively examined to determine if these nanomaterials themselves could demonstrate adverse health effects, especially if used long-term by patients. Indeed, since the composing chemical(s) may or may not be soluble in biological matrices, the toxicology of applied nanocomposites may differ greatly from that for loaded bioactive substances; it may substantially influence outcomes for different internal organs (De Jong and Borm, 2008). For several nanoantioxidant formulations, a burst drug release may result in concerns related to cellular toxicity, while a slower drug release, on the contrary, may lead to insufficient therapeutic efficacy. Therefore, the development of nanoformulations with release profiles optimized according to the physicochemical properties of carried therapeutic agents presents an essential research challenge that bears further investigation (Lin et al., 2017). Given these considerations, outstanding questions also include: (1) whether applied nanomaterials might bioaccumulate in the human body? (2) Whether they may be converted into potentially dangerous metabolites? (3) Whether they can be completely degraded and subsequently excreted after delivering the therapeutic agent? (4) Whether they can become hazardous after being excreted (in urine or feces of treated patients) and accumulated in living human environments? Given these remaining challenges, it may be concluded that, even though essential steps have been made to bringing nanotherapeutic approaches closer to clinical applications, additional investigation is needed to improve the effectiveness and long-term safety of bioactive compound-loaded nanodelivery systems in therapy of aging-associated disorders.

## Author Contributions

AV, AK, AZ, and OL conceptualized and designed the manuscript. AV performed literature search and wrote the first draft of the manuscript. AK, AZ, and OL edited the manuscript and constructed the figures. The manuscript was written through contributions of all authors. All authors approved the final version of the manuscript.

### Conflict of Interest

The authors declare that the research was conducted in the absence of any commercial or financial relationships that could be construed as a potential conflict of interest.
